# The Expression of microRNA in Adult Rat Heart with Isoproterenol-Induced Cardiac Hypertrophy

**DOI:** 10.3390/cells9051173

**Published:** 2020-05-08

**Authors:** Mailin Gan, Shunhua Zhang, Yuan Fan, Ya Tan, Zhixian Guo, Lei Chen, Lin Bai, Dongmei Jiang, Xiaoxia Hao, Xuewei Li, Linyuan Shen, Li Zhu

**Affiliations:** 1 College of Animal Science and Technology, Sichuan Agricultural University, Chengdu 611130, China; ganmailin@stu.sicau.edu.cn (M.G.); 14081@sicau.edu.cn (S.Z.); fanyuan@stu.sicau.edu.cn (Y.F.); tanya@stu.sicau.edu.cn (Y.T.); 2018302019@stu.sicau.edu.cn (Z.G.); chenlei815918@sicau.edu.cn (L.C.); blin16@126.com (L.B.); jiangdm@sicau.edu.cn (D.J.); xiaoxia6363@126.com (X.H.); xuewei.li@sicau.edu.cn (X.L.); 2 Farm Animal Genetic Resource Exploration and Innovation Key Laboratory of Sichuan Province, Sichuan Agricultural University, Chengdu 611130, China; 3 Institute of Animal Husbandry and Veterinary, Guizhou Academy of Agricultural Science, Guiyang 550005, China

**Keywords:** cardiac hypertrophy, miRNAs, miR-144, LncMIAT, isoproterenol

## Abstract

Cardiac hypertrophy is a common pathological condition and an independent risk factor that triggers cardiovascular morbidity. As an important epigenetic regulator, miRNA is widely involved in many biological processes. In this study, miRNAs expressed in rat hearts that underwent isoprenaline-induced cardiac hypertrophy were identified using high-throughput sequencing, and functional verification of typical miRNAs was performed using rat primary cardiomyocytes. A total of 623 miRNAs were identified, of which 33 were specifically expressed in cardiac hypertrophy rats. The enriched pathways of target genes of differentially expressed miRNAs included the FoxO signaling pathway, dopaminergic synapse, Wnt signaling pathway, MAPK (mitogen-activated protein kinase) signaling pathway, and Hippo signaling pathway. Subsequently, miR-144 was the most differentially expressed miRNA and was subsequently selected for in vitro validation. Inhibition of miR-144 expression in primary myocardial cells caused up-regulation of cardiac hypertrophy markers atrial natriuretic peptide (ANP) and brain natriuretic peptide (BNP). The dual luciferase reporter system showed that ANP may be a target gene of miR-144. Long non-coding RNA myocardial infarction associated transcript (LncMIAT) is closely related to heart disease, and here, we were the first to discover that LncMIAT may act as an miR-144 sponge in isoproterenol-induced cardiac hypertrophy. Taken together, these results enriched the understanding of miRNA in regulating cardiac hypertrophy and provided a reference for preventing and treating cardiac hypertrophy.

## 1. Introduction

Cardiac hypertrophy is a compensatory response of the heart to provide normal cardiac output under a variety of cardiovascular diseases, including hypertension and coronary heart disease [1]. Although cardiac hypertrophy is a powerful type of compensation, if the cause cannot be eliminated for a prolonged period of time, the function of the hypertrophic myocardium cannot remain normal for a long time and eventually turns to heart failure [2]. In previous studies, it has been shown that heart failure affects 2% of the global adult population [3]. Preventing and reversing cardiac hypertrophy in patients with cardiovascular disease can significantly improve patient prognosis.

Cardiac hypertrophy involves a variety of pathophysiological signals, such as physical, chemical, and hormonal levels, including angiotensin II (Ang II), norepinephrine and beta-adrenergic receptor agonists [4,5]. Isoprenaline (ISO) is a beta-receptor agonist that is commonly used in the treatment of bronchial asthma and cardiac atrioventricular block [6,7]. Previous studies have shown that ISO can trigger cardiac hypertrophy by activating atrial natriuretic peptide (ANP) and brain natriuretic peptide (BNP) [8]. ANP is mainly secreted by atrial myocytes, and BNP is mainly synthesized and secreted by ventricular myocytes [9,10]. During cardiac hypertrophy, ANP and BNP are secreted in large amounts [11]. Therefore, ANP and BNP are often used as markers of cardiac hypertrophy [12].

Epigenetics mainly studies the interaction between environment and genes and reveals the influence of environment on biological genetic material. MicroRNA (miRNA) is a widely studied epigenetic regulatory factor that is involved in the regulation of post-transcriptional gene expression in plants and animals [13]. In previous studies, it has been shown that miRNAs are involved in cardiac hypertrophy caused by various underlying conditions. However, the regulation of miRNAs on cardiac hypertrophy is still not fully understood [14]. Linear non-coding RNAs with more than 200 nucleotides are defined as long non-coding RNAs (LncRNAs). LncRNAs can affect gene expression and regulate molecular signaling pathways in various ways [15]. Long non-coding RNA myocardial infarction associated transcript (LncMIAT) was initially found to be related to myocardial infarction [16]. An important function of LncRNAs is as endogenous competitive RNAs in combination with miRNAs to regulate gene expression. LncMIAT has been reported to affect the heart disease process by combining miR-22 [17] and miR-379 [18]. With the deepening of the research, LncMIAT was also found to be related to myocardial hypertrophy, but its molecular mechanism and regulatory network have not been fully resolved.

In the present study, we focused on the possible functional role of miRNAs in ISO-induced cardiac hypertrophy. The results of this study may provide novel insights and references for the prevention and treatment of cardiac hypertrophy. 

## 2. Materials and Methods

### 2.1. Ethics Statement

Animal care and procedures were conducted in accordance with the requirements of the United Kingdom Animals (Scientific Procedures) Act 1986. The experimental protocol was approved by the Animal Care and Ethics Committee of Sichuan Agricultural University (Sichuan, China, No. DKY-B20131403). 

### 2.2. Animals and Treatment

Male Sprague Dawley (SD) rats (8 weeks, 286.83 ± 7.81 g) were purchased from Chengdu Dashuo Experimental Animal Co, Ltd. (Chengdu, China). Rats were housed in standard plastic cages under a controlled temperature (22 ± 3 °C) and a natural light cycle. Animals were given free access to food and water. After 10 days of environmental adaptation, a model of cardiac hypertrophy was induced by intraperitoneal injection of ISO (5 mg/kg·day) continuously for 14 days [19]. The control group received the same amount of physiological saline solution (NC, 0.9% NaCl).

### 2.3. Small RNA Sequencing 

Total RNA extracted from the heart of ISO-induced cardiac hypertrophy rats was used for miRNA sequencing. Small RNA was purified by 15% denatured polyacrylamide gel electrophoresis (PAGE), and 15–40 nt RNA was isolated from total RNA. This was used to construct a small RNA sequence library and was sequenced on the Illumina HiSeq 2000 device. 

### 2.4. Analysis of Sequencing Data

The flow of small RNA-seq data processing and analysis was performed as described previously [20]. First, low-quality reads and adaptor sequences were removed to obtain clean reads. Subsequently, the Burrows–Wheeler alignment (BWA) mapping tool was used to map clean data to the rat reference genome [21]. To assess the differentially expressed miRNAs, the DESeq2 package in the statistics software R (Version 3.0.1) was used with standard parameters. To avoid false positive results, miRNAs of |Log2(Fold change)| ≥ 1 were verified by real-time quantitative PCR (RT-qPCR).

### 2.5. Target Prediction and Functional Annotation of Target Genes

TargetScan [22] (http://www.targetscan.org/) and miRBD [23] (http://mirdb.org/) were used to predict the target genes of miRNAs (Top 10 and |Log2(Fold change)| ≥ 1). For more accurate results, only target genes predicted by both programs were retained. GO: cellular component, molecular function, and biological process and Kyoto Encyclopedia of Genes and Genomes (KEGG) analyses were performed on the predicted target genes [24] (https://david.ncifcrf.gov/conversion.jsp?VFROM=NA).

### 2.6. Real-Time Quantitative PCR

Total RNA was extracted from rat hearts and cells using TRIzol reagent (Invitrogen, Guangzhou, China). MiRNA RT-qPCR Primer Set (TsingKe, Chendu, China) was used to determine the expression levels of miRNAs by RT-qPCRs SYBR Premix Ex Taq kit (TaKaRa, Dalian, China) in a BioRad CFX96 Real-Time PCR Detection System (Bio-Rad, Richmond, CA, USA). U6 was used as an miRNA internal control, and GAPDH (glyceraldehyde-3-phosphate dehydrogenase) served as an mRNA internal control. TPprimer sequences are listed in Supplementary Table S1. 

### 2.7. Cell Culture and Transfection

Rat myocardial primary cells were derived from a litter of newborn SD rat hearts according to a previous report with adjustments [25,26]. Isolated rat primary cardiomyocytes are shown in Supplementary Video S1. Rat myocardial primary cells were seeded in 12-well plates. When the cell confluence reached about 50%, miR-144 mimic (50 nM), inhibitor (50 nM), siLncMIAT (50 nM), ISO (10 μM), and the negative control (Ribobio, Guangzhou, China) were transfected into rat myocardial primary cells using Lipofectamine 3000 (Invitrogen, Guangzhou, China). Cells were harvested 24 h after transfection.

### 2.8. Dual Luciferase Reporting System

The dual luciferase reporting system involved amplification of the 3′UTR of ANP and LncMIAT fragments containing an miR-144 target site by PCR, and insertion into the psiCHECK™-2 vector. The psiCHECK™-2 vector and the smiR-144 mimic were co-transfected into HeLa cells using Lipofectamine 3000. Cells were collected after 48 h of transfection and luciferase activity was measured using the Dual-Glo Luciferase Assay System (Promega, Madison, WI, USA) following the manufacturer’s instructions.

### 2.9. Statistical Analysis 

Results are presented as the mean ± s.e.m. The Student’s *t*-test was employed for comparisons between two groups. One-way ANOVA analysis was performed where there were more than two groups. Statistical analyses were performed using SPSS 22.0 software. *p* ≤ 0.05 was considered significant.

## 3. Results

### 3.1. Isoproterenol-Induced Cardiac Hypertrophy in Rats

**Figure 1:**
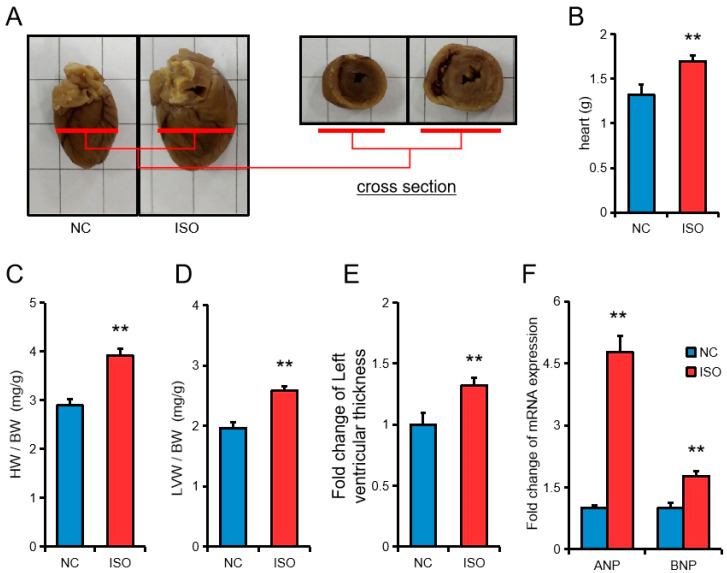
Isoproterenol induces rat cardiac hypertrophy in vivo. (**A**) Images of the whole hearts and cross section of Sprague Dawley (SD) rats. (**B**,**C**) Heart weight (**B**) and the ratio of heart weight to body weight (**C**) in rats. (**D**) The ratio of left ventricular weight to body weight. (**E**) Fold change of left ventricular thickness in rats. (**F**) Fold change of RNA expression in rats. All results are presented as the mean ± SEM. *n* = 6. * *p* < 0.05, ** *p* < 0.01.

Cardiac hypertrophy in rats was successfully induced by subcutaneous injection of isoproterenol. After isoproterenol (ISO) injection, the lateral diameter of the heart increased to 1.3-fold compared to that of the control group, and the long diameter increased to 1.2-fold compared to that of the control group (Figure 1A). Compared with the control group, the cardiac weight, cardiac index, LVW/BW ratio (LVW: left ventricular weight; BW: body weight) and left ventricular thickness of the ISO group increased significantly (Figure 1B–E). Injection of ISO also significantly increased the expression of ANP and BNP, which are markers of cardiac hypertrophy (Figure 1F).

### 3.2. Overview of microRNAs Sequencing Data

To determine the characteristics and abundance of miRNAs during the process of ISO-induced cardiac hypertrophy in rats, the miRNA profiles of hearts of normal rats and cardiac hypertrophy rats were sequenced. As shown in Table 1, an average of 29237702 and 25571903 raw readings were obtained from the normal control group (NC) and cardiac hypertrophy group (ISO), respectively. In addition, the clean reads Q30 values of sequenced samples all exceeded 95%. 

### 3.3. Expression Character Analysis of miRNAs in Rat Myocardium

**Figure 2:**
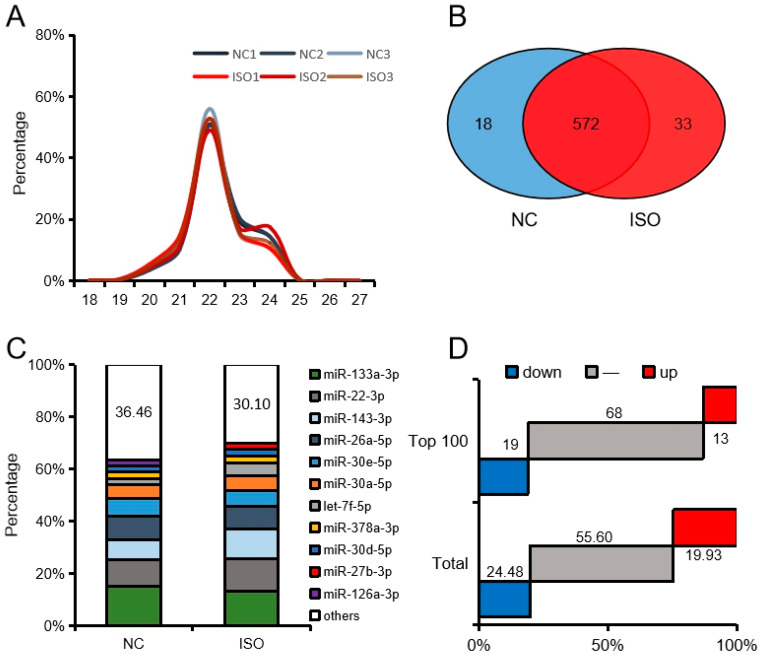
Expression characteristics of rat myocardial miRNA. (**A**) Size distribution of miRNAs. (**B**) The Venn diagrams represent the number of expressed miRNAs. (**C**) Composition of highly expressed miRNAs. (**D**) Differential expression of miRNAs (down: Fold change ≤ 2/3, up: Fold change ≥ 3/2).

Further analysis revealed that the sequence length was between 20 and 24 nucleotides (nt), which accounted for 99.18%, and the 22 nt read had the highest abundance and accounted for 52.03% (Figure 2A). A total of 623 miRNAs were identified, and 572 were detected in both NC and ISO groups (Figure 2B). The top 10 miRNAs accounted for 63.54% in the NC group, and 69.90% in the ISO group. In addition, in the NC group and ISO group, nine miRNAs were the same among the Top 10 miRNAs (Figure 2C). We used ISO / NC as the standard (down: Fold change ≤ 2/3, up: Fold change ≥ 3/2) and found that in all miRNAs, 24.48% were up-regulated and 19.93% down-regulated. Among the top 100 miRNAs, 13 were up-regulated and 19 were down-regulated (Figure 2D).

### 3.4. RT-qPCR Validation of the Differentially Expressed miRNAs

**Figure 3:**
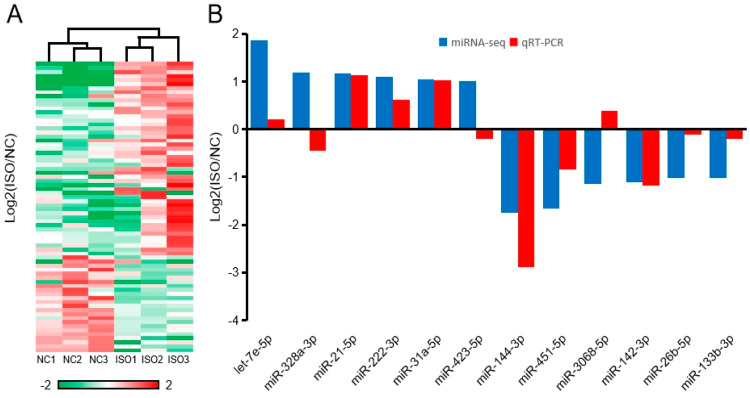
Validation of differentially expressed miRNAs by qRT-PCR. (**A**) The heat maps show the expression of miRNAs detected by sequencing. (**B**) The expression of miRNAs (Among the top 100 highly expressed miRNAs, Fold change ≥ 2 or Fold change ≤ 0.5) detected by RT-qPCR.

Cluster analysis of differentially expressed miRNAs showed that three samples from the NC group were clustered into one branch, while three samples from the ISO group were clustered into another branch (Figure 3A). As shown in Figure 3B, in the top 100 expression levels, six miRNAs were up-regulated (Fold change ≥ 2) and six were down-regulated (Fold change ≤ 0.5). RT-qPCR verified that from only three miRNAs (miR-328a-3p, miR423-5p, and miR-3068-5p), the expression trend did not match the sequencing results (Figure 3B).

### 3.5. miRNA Target Prediction, GO, and KEGG Pathway Analyses

**Figure 4:**
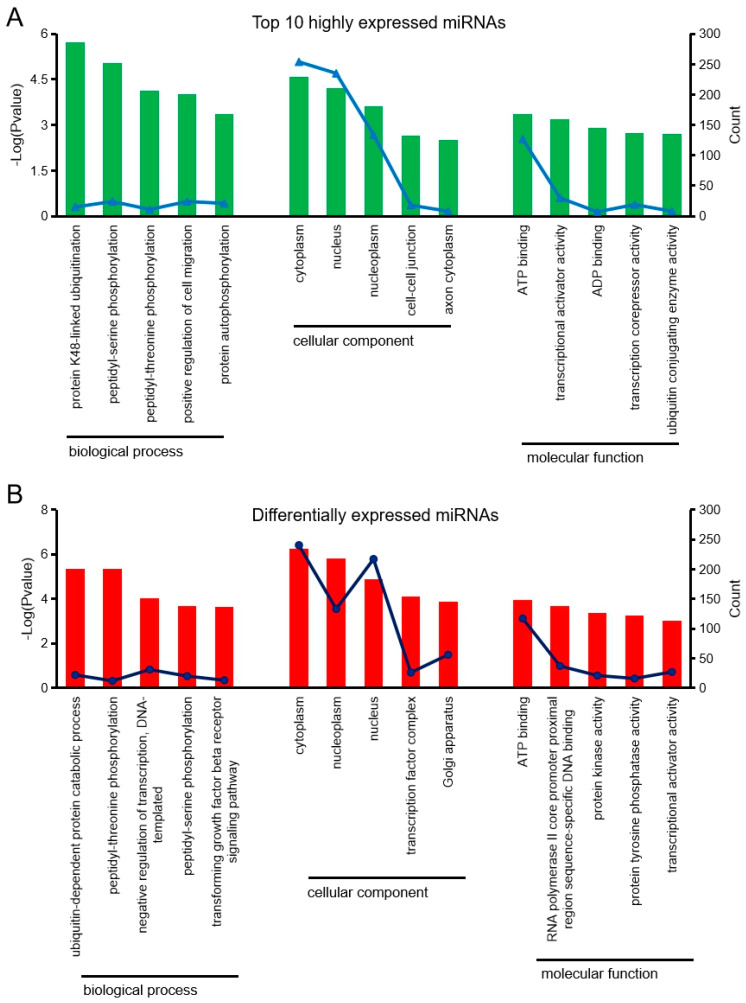
Partial gene ontology classification for predicted miRNAs. (**A**) Gene ontology-biological processes enrichment of target genes for top 10 highly expressed miRNAs. (**B**) Gene ontology–biological processes enrichment of target genes for differentially expressed miRNAs (RT-qPCR validated miRNAs).

**Figure 5:**
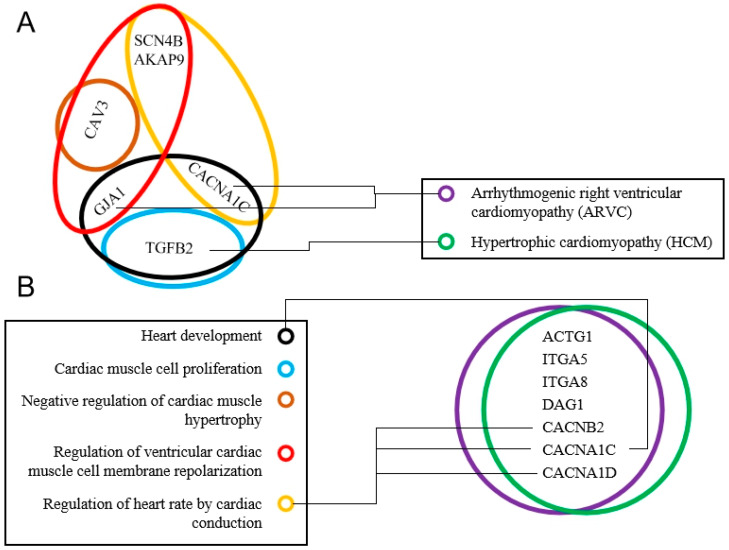
Heart-related signaling pathways. (**A**) Repeated genes in different heart-related biological processes in heart-related signaling pathways. (**B**) Repeated genes in different cardiac-related signaling pathways in cardiac-related biological processes. The same color on the left side of (**A**,**B**) represents the same signaling pathways; the same color on the right side of (**A**,**B**) represents the same biological processes.

To perform biological functions, miRNAs rely on their target genes. miRNA target gene prediction software was used to predict the target genes of the top 10 highly expressed miRNAs and the differentially expressed miRNAs, and gene ontology (GO) enrichment analysis of these putative target genes was performed. Target gene prediction revealed that 1331 genes were targeted by the top 10 highly expressed miRNAs, while 1185 genes were targeted by nine differentially expressed miRNAs. Analysis of the biological processes of the top 10 highly expressed miRNAs and the differentially expressed miRNAs showed that the most significant gene enrichment involved the ubiquitination process. Analysis of the cellular components showed that most genes were enriched in the cytoplasm. In addition, analysis of molecular function showed that most genes were clustered into binding activities (Figure 4A,B). Furthermore, target genes for differentially expressed miRNAs were also enriched during heart development, cardiac muscle cell proliferation, negative regulation of cardiac muscle hypertrophy, regulation of ventricular cardiac muscle cell membrane repolarization, and the regulation of heart rate by cardiac conduction, which are all cardiac-related biological processes (Figure 5A,B).

Kyoto Encyclopedia of Genes and Genomes (KEGG) pathway annotation showed that 47 and 68 biological functions were annotated by target genes of the top 10 highly expressed miRNAs and differentially expressed genes, respectively. The top 10 highly expressed miRNAs were involved in miRNAs in cancer, axon guidance, MAPK (mitogen-activated protein kinase) signaling pathway, pathways in cancer, enrichment pathways of differentially expressed miRNAs of target genes, including FoxO signaling pathway, dopaminergic synapse, Wnt signaling pathway, MAPK signaling pathway, and the Hippo signaling pathway. In addition, 12 and 10 target genes were significantly enriched in arrhythmogenic right ventricular cardiomyopathy (ARVC) and hypertrophic cardiomyopathy (HCM) signaling pathways, respectively (Supplementary Table S2, Figure 5A,B).

### 3.6. miR-144 Regulates Isoproterenol-Induced Cardiac Hypertrophy by Targeting ANP

Among these differently expressed microRNAs, sequencing results and RT-qPCR showed that changes in the expression miR-144 were the largest. KEGG analysis of target genes of miR-144 revealed that its enrichment pathways were mainly Wnt signaling pathway, cGMP-PKG signaling pathway, FoxO signaling pathway, TGF-beta signaling pathway, and AMPK signaling pathway, which were highly coincident with differentially expressed miRNA enrichment signaling pathways (Supplementary Table S3). Taken together, these analyses indicated that miR-144 had a broad representative role in ISO-induced cardiac hypertrophy differentially expressed miRNAs. 

**Figure 6:**
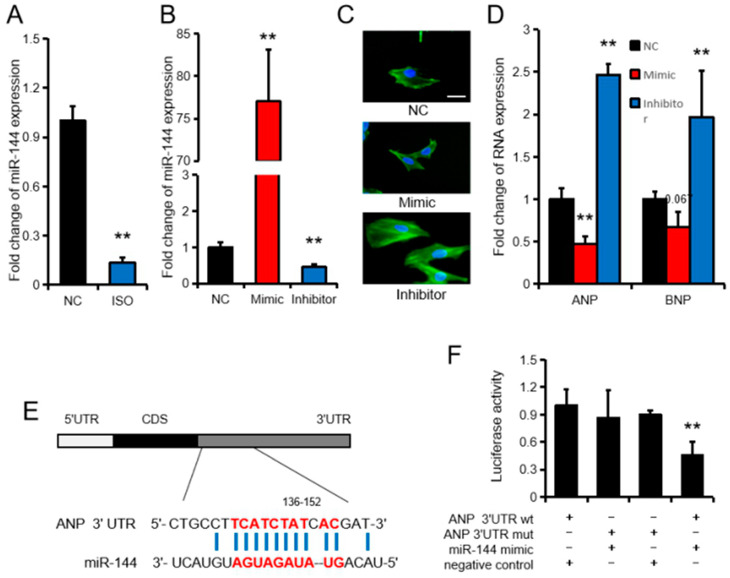
miR-144 regulates cardiac hypertrophy by targeting atrial natriuretic peptide (ANP). (**A**) The expression of miR-144 in rat myocardial primary cells after treatment with 10 μM isoprenaline (ISO). (**B**,**D**) Fold change of RNA expression. MiR-144 (**B**), ANP, and brain natriuretic peptide (BNP) (**D**) expression in rat myocardial primary cells after transfection with the miR-144 mimic, inhibitor, or negative control. (**C**) Rat myocardial primary cells were transfected for 24 h, as indicated above. Cellular F-actin and the nuclei of the cell in each group were stained with FITC-phalloidin (fluorescein isothiocyante-phalloidin) and DAPI (4′,6-diamidino-2-phenylindole). The bars of the charts indicate 25 µm. (**E**) Binding site of miR-144 and ANP. (**F**) HeLa cells were co-transfected psiCHECKTM-2 vectors and the miR-144 mimic or a negative control, and luciferase activity was determined. (**B**–**D**) are all carried out under normal conditions, without ISO treatment. Results are presented as the mean ± SEM. *n* = 3. * *p* < 0.05, ** *p* < 0.01.

As an epigenetic regulatory factor, miRNAs rely on their target genes to perform their biological functions. miR-144 was found to be the miRNA with the largest change in differential expression among the top 100 miRNAs both in sequencing and RT-qPCR data (Figure 3B). Therefore, we chose miR-144 for in vitro validation at the cellular level. Our data showed that the expression of miR-144 in rat myocardial primary cells was significantly down-regulated (*p* < 0.01) after treatment with 10 μM ISO (Figure 6A). Furthermore, by transfecting an miR-144 mimic and miR-144 inhibitor into the myocardial primary cells, we successfully overexpressed or suppressed miR-144 expression (Figure 6B,C). Inhibition of miR-144 in rat myocardial primary cells significantly increased (*p* < 0.01) expression of the cardiac hypertrophy marker genes ANP and BNP. However, overexpression of miR-144 significantly inhibited (*p* < 0.01) the expression of ANP (Figure 6D). Interestingly, we found that miR-144 had potential binding sites to the 3’UTR of ANP (Figure 6E). The results of a dual luciferase reporter system further confirmed the binding of ANP and miR-144. When compared with transfection of the negative control and mutant psiCHECKTM-2 vectors (ANP 3’UTR mut), the luciferase activity was significantly decreased (*p* < 0.01) after transfection of wild-type psiCHECKTM-2 vectors (ANP 3’UTR wt) and an miR-144 Mimic (Figure 6F). 

### 3.7. miR-144 Regulates Cardiac Hypertrophy by Targeting ANP and is Regulated by LncMIAT

**Figure 7:**
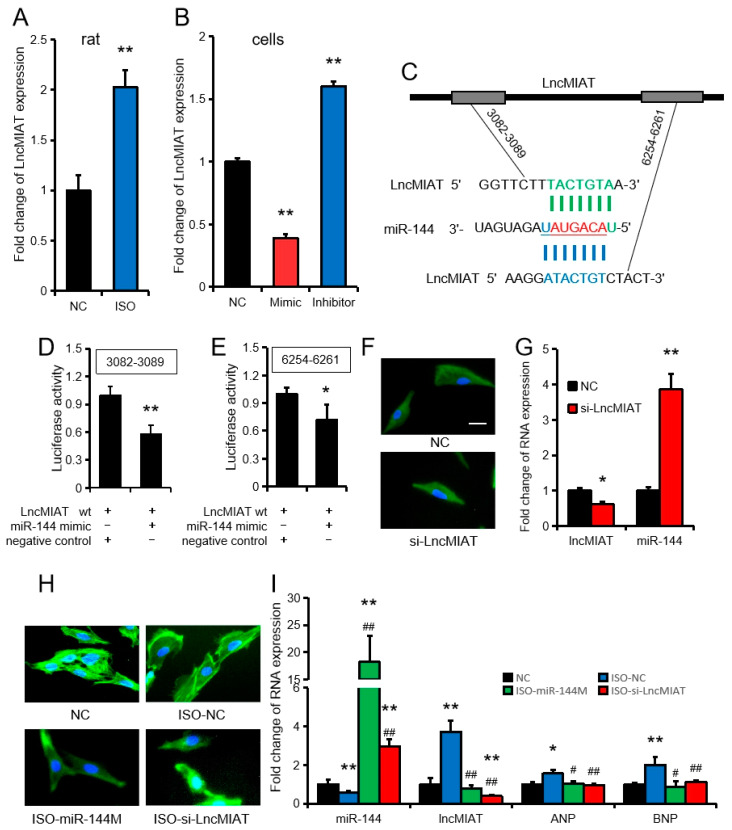
Long non-coding RNA myocardial infarction associated transcript (LncMIAT) acts as an miR-144 sponge and is involved in cardiac hypertrophy. (**A**,**B**) The expression of LncMIAT in rat myocardial primary cells after treatment with 10 μM isoprenaline (ISO) (**A**) or transfection with the miR-144 mimic, inhibitor, or negative control (**B**). (**C**) Binding site of miR-144 and LncMIAT. (**D**–**E**) The fluorescence intensity of the dual luciferase reporter assay. (**F**) Rat myocardial primary cells were transfected for 24 h, as indicated above. Cellular F-actin and the nuclei of the cell in each group were stained with FITC-phalloidin and DAPI. The bars of the charts indicate 25 µm. (**B**,**D**–**F**) are all carried out under normal conditions, without ISO treatment. (**G**) The expression of LncMIAT and miR-144 in rat myocardial primary cells after transfection with siRNA (si-LncMIAT) or the negative control (NC). (**H**) Rat myocardial primary cells were transfected for 24 h, as indicated above. Cellular F-actin and the nuclei of the cell in each group were stained with FITC-phalloidin and DAPI. The bars of the charts indicate 25 µm. (**I**) Fold change of RNA expression in rat myocardial primary cells after transfection with “negative control (NC)”, “negative control + ISO (ISO-NC)”, “miR-144 Mimic + ISO (ISO-miR-144M)”, and “si-LncMIAT + ISO (ISO-si-LncMIAT)”. All results are presented as the mean ± SEM. n = 3. * *p* < 0.05, ** *p* < 0.01, when compared to the negative control (NC). # *p* < 0.05, ## *p* < 0.01 when compared to the ISO-treated group.

LncMIAT is a well-known LncRNA that regulates cardiac hypertrophy. In this study, LncMIAT expression was significantly increased (*p* < 0.01) after treatment with ISO, both in vitro and in vivo (Figure 7A,G). Overexpression of miR-144 in rat primary cardiomyocytes significantly inhibited LncMIAT expression (*p* < 0.01), while inhibition of miR-144 significantly promoted LncMIAT expression (*p* < 0.01) (Figure 7B). It is worth noting that when the identified five down-regulated miRNAs (Figure 3B) were analyzed, a potential binding relationship between three miRNAs and LncMIAT was found (Supplementary Figure S1), and that two miR-144 binding sites in LncMIAT were detected (Figure 7C). Furthermore, the results from the dual luciferase reporter system proved that both binding sites had binding relationships (Figure 7D,E). Using small interfering RNA (siRNA) technology, we successfully suppressed LncMIAT expression and determined that miR-144 was up-regulated in rat primary myocardial cells (Figure 7F,G). Co-transfection results showed that the miR-144 mimic and si-LncMIAT significantly reduced the expression of ANP and BNP in ISO-induced rat primary myocardial cells (Figure 7H,I). 

## 4. Discussion

Cardiac hypertrophy is the response of the heart to long-term stress or volume loads [27]. In this state, myocardial cells gradually enlarge, and at the same time, a series of changes, including hormone levels, gene expression, and cell structure occur [28]. If this series of changes cannot be improved in a short period of time, this will lead to heart expansion, myocardial contraction, and diastolic function decrease, thereby leading to heart failure [3]. MicroRNA (miRNA) is a type of non-coding RNA with a length of about 22 nt. It is widely present in eukaryotic cells and plays an important role in many physiological and pathological processes, including animal and plant growth, development, reproduction, and disease occurrence [29]. Here, we attempted to identify miRNA characteristics of the hearts of normal and cardiac hypertrophy rats by using sequencing techniques. In addition, functional verification and regulatory network construction of typical miRNAs were performed in vitro. These findings will help us to further understand the cellular functions and pathophysiology of miRNAs in the heart.

### 4.1. The miRNA Characteristics of Isoprenaline-Induced Cardiac Hypertrophy

High-abundance miRNAs may play an important role in maintaining normal myocardial function. Our data showed that the top 10 highly expressed miRNAs in normal hearts were miR-133a-3p, miR-22-3p, miR-26a-5p, miR-143-3p, miR-30e-5p, miR-30a-5p, miR-378a-3p, let-7f-5p, miR-30d-5p, and miR-126a-3p, and that the top 10 miRNAs expressed in hypertrophic hearts were miR-133a-3p, miR-22-3p, miR-143-3p, miR-26a-5p, miR-30e-5p, miR-30a-5p, let-7f-5p, miR-378a-3p, miR-30d-5p, and miR-27b-3p. Interestingly, 9 of the top 10 highly expressed miRNAs in the two groups were identical, and the cumulative ratio was more than 60% of the total miRNA. These miRNAs were found to be similarly expressed in other studies [30]. Among them, miR-133a-3p [31], miR-22-3p [32], miR-143-3p [33], miR-26a-5p [34], let-7f-5 p [35], miR-378a-3p [36], miR-27b-3p [37], and the miR-30 family [38] have all been reported to be involved in the occurrence of cardiac diseases. These results imply that the expression and function of highly abundant miRNAs in different physiological states are conservative.

Differential expression analysis of sequencing data revealed that let-7e-5p, miR-328a-3p, miR-21-5p, miR-222-3p, miR31a-5p, and miR-423-5p were up-regulated, and that miR-144-3p, miR-451-5p, miR-3068-5p, miR-142-3p, miR-26b-5p, and miR-133b-3p were down-regulated. Rt-qPCR showed that the expression trends of miR-328a-3p, miR-423-5p, and miR-3068-5p were not consistent with the sequencing results. In previous studies, it was reported that let-7e-5p and miR-21-5p were upregulated in patients with heart failure [39]. Furthermore, miR-21-5p, miR-31-5p, and miR-222-3p were up-regulated in afterload enhanced or enhancement (AE)-induced myocardial pathological hypertrophy in rats [31]. MiR-142-3p was down-regulated in the abdominal aorta (AB) and AngII-induced cardiac hypertrophy in rats [40]. MiR-26-5p was down-regulated in exercise-induced cardiac hypertrophy in rats [41]. MiR-133b was down-regulated in MHCα-CN murine heart failure model [42]. MiR-451 was also found to be down-regulated in isoproterenol-induced cardiac hypertrophy [8] and patients with heart failure [39] but was up-regulated in high-fat diet-induced cardiac hypertrophy [43]. Little is known about the role of miR-144-3p in cardiac hypertrophy. However, these results suggest that there are some differences in miRNA expression characteristics in cardiac hypertrophy induced by different factors.

Functional enrichment analysis of high-abundance miRNAs and differentially-expressed miRNAs showed that high-abundance miRNAs are mainly concentrated in miRNAs in cancer, axon guidance, the MAPK signaling pathway, pathways in cancer, the FoxO signaling pathway, and the Wnt signaling pathway. Axon guidance signaling pathways are related to neurodevelopment [44]. The MAPK signaling pathway, FoXO signaling pathway, and Wnt signaling pathway are conserved in mammals and play important biological functions. MAPKs are serine/threonine protein kinases that mediate the response of a cell to a variety of extracellular stimuli [45]. The FoxO signaling pathway is involved in cell proliferation [46], and Wnt pathways are involved in the control of gene expression, cell behavior, cell adhesion, and cell polarity [47]. Taken together, these results and previous reports [48] have suggested that MAPK, FoxO, and Wnt signaling pathways are of great significance for normal physiological functioning of cardiomyocytes. 

Differentially expressed miRNAs are mainly concentrated in the FoxO signaling pathway, dopaminergic synapse, Wnt signaling pathway, MAPK signaling pathway, and the Hippo signaling pathway. It is worth noting that among the differentially expressed miRNA enrichment pathways, the five most significant pathways are dopaminergic synapses and the Hippo signaling pathway that are different from high-abundance miRNA enrichment pathways. The Hippo signaling pathway is highly conserved among different species and has been reported to be related to important processes, including organ size, cancer occurrence, tissue regeneration, and stem cell function [49,50]. Moreover, Hippo signaling has been reported to play a role in cardiac hypertrophy induced by multiple factors [51,52]. Interestingly, dopamine is a precursor of norepinephrine and adrenaline synthesis [53]. Therefore, it is not difficult to understand that the differential miRNAs of the heart after ISO treatment are significantly enriched in the dopaminergic synapse. Thus, the dopaminergic synapse may play a unique role in ISO-induced cardiac hypertrophy.

Differentially expressed miRNAs were not the same as the top 10 miRNAs, but functional enrichment analysis showed that their enrichment pathways and biological processes were highly overlapping. These same biological processes and signaling pathways were likely to be common features in the process of cardiac hypertrophy. Key molecules involved in these signaling pathways can be used as markers to identify cardiac hypertrophy, and the specifically expressed molecules can help us to analyze the specific cause of cardiac hypertrophy.

### 4.2. New Molecular Mechanism of Isoprenaline-Induced Cardiac Hypertrophy

In previous studies, it was indicated that miR-144 was associated with heart disease [54,55]. However, research on its function in cardiac hypertrophy is still lacking [56]. Here we found that miR-144 was downregulated in ISO-induced cardiac hypertrophy, both in vivo and in vitro. Furthermore, transfection of an miR-144 inhibitor inhibited miR-144 expression in primary cardiomyocytes, and the expression of cardiac hypertrophy marker genes ANP and BNP was up-regulated in primary cardiomyocytes. To quickly identify a research direction and focus on a large amount of data, typical functional analysis methods are warranted.

When compared with genes, including Connexin 43 (COX43) [57] and Rac1 [54] that have been predicted and experimentally verified for binding, we found a potential binding relationship between miR-144 and ANP through sequence analysis. We verified the binding relationship between miR-144 and ANP using the dual luciferase reporter system. Target genes that depend on miRNA seed sequences (nucleotides 2-7) have been extensively studied [58], and recent studies have revealed the important role of miRNA sequences in the recognition and regulation of specific targets outside the seed sequence [59]. Breaking through traditional and comprehensive analysis when conducting specific mechanism research is more conducive to the interpretation of the problem and the in-depth knowledge of objective facts. In addition to miR-144 inhibiting its target gene expression through strict seed sequence matching targeting, non-classical binding to ANP may have important significance in cardiac hypertrophy. 

LncRNAs have been reported to be competitive endogenous RNAs by sponging miRNAs. As a bridge for LncRNA to play a biological function, miRNA has an important role in the functional regulation of LncRNA [60]. Previous studies have found that LncRNA such as lncMIAT [61], lncHoTAIR [62], LncROR [63] and LncH19 [64] are closely related to cardiac hypertrophy. Among them, LncMIAT is one of the most extensive LncRNAs in the study of cardiac hypertrophy [65]. In addition, through sequence analysis, we found that LncMIAT has a potential binding relationship with four of the six miRNAs, where expression was down-regulated in the sequencing study (Supplementary Figure S1). It is worth noting that lncMIAT has two binding sites for miR-144 and miR-133, and the binding relationship between miR-133 and LncMIAT has previously been reported [66]. Therefore, we verified the binding relationship between miR-144 and LncMIAT using the dual luciferase reporter assay. In addition, primary in vitro co-treatment experiments initially showed that both overexpression of miR-144 and interference with LncMIAT can partially alleviate ISO-induced cardiac hypertrophy. 

**Figure 8:**
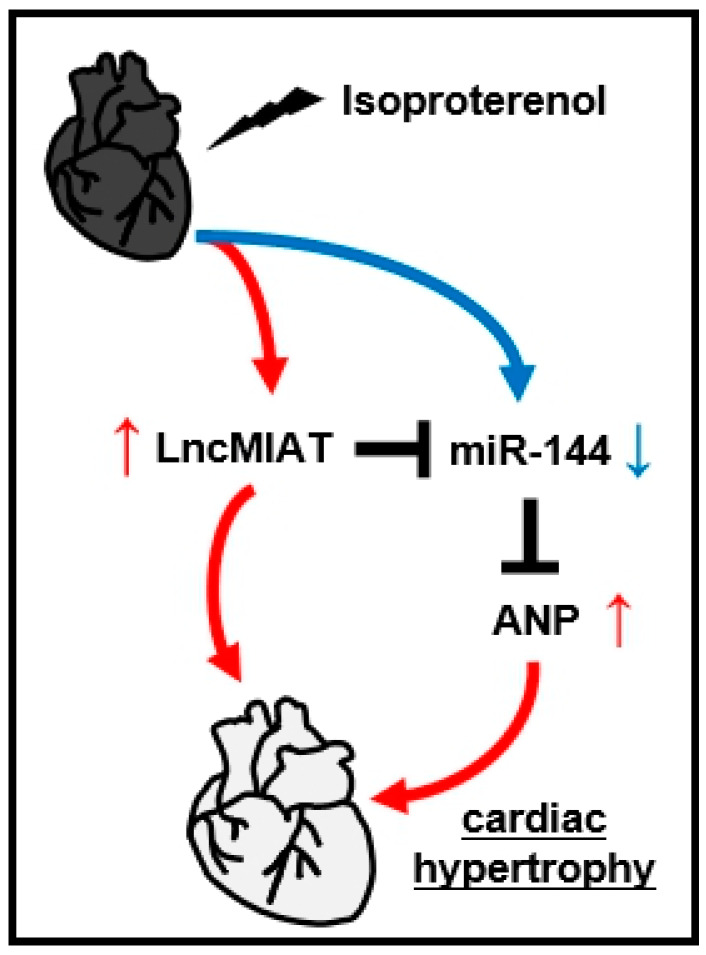
LncMIAT participates in isoproterenol-induced cardiac hypertrophy through miR-144-ANP.

According to our results, a new molecular network that regulates cardiac hypertrophy is gradually emerging: ISO → LncMIAT → miR-144 → ANP → cardiac hypertrophy (Figure 8).

## 5. Conclusions

In summary, miRNAs are abnormally expressed in ISO-induced cardiac hypertrophy. Among them, let-7e-5p, miR-21-5p, miR-222-3p, and miR31a-5p were up-regulated, while miR-144-3p, miR-451-5p, miR-142-3p, miR-26b-5p, and miR-133b-3p expression were down-regulated. Combined with the data presented in previous reports, the MAPK signaling pathway, FoxO signaling pathway, and Wnt signaling pathway play an important role in the normal physiological function of the heart, and the dopaminergic synapse may be a signaling pathway that is involved in ISO-induced cardiac hypertrophy. In addition, both differential expression and functional enrichment analysis showed that miR-144 was a representative miRNA in ISO-induced cardiac hypertrophy. Furthermore, we improved the “ISO → LncMIAT → miR-144 → ANP → cardiac hypertrophy” regulatory network, which will further help us to understand the function of miR-144 in cardiac hypertrophy. Collectively, our results contributed to a better understanding of the role of miRNAs in cardiac hypertrophy and provide a reference for the prevention and treatment of cardiac hypertrophy.

## Figures and Tables

**Table 1:** Summary of reads from raw data and clean reads for microRNAs (miRNAs) sequencing in rat hearts.

Sample	Control Group			ISO Group		
	NC-1	NC-2	NC-3	ISO-1	ISO-2	ISO-3
Total Raw Reads	26418839	32847346	28446923	31732589	22959111	22024011
Low Quality Reads	436	600	605	477	726	738
Poly A/T Rate (%)	5.09	4.54	3.75	5.29	4.53	4.69
Ex-length Rate (%)	1.38	1.44	0.78	3.8	1.45	1.44
Clean Reads Rate (%)	90.71	90.31	91.23	87.85	87.4	86.59
Clean Reads Q30 (%)	98.48	98.42	98.09	98.58	95.58	95.25
Mapping Rate (%)	93.95	93.49	97	93.75	86.23	86.9

Abbreviations: ISO: isoproterenol; NC: normal control.

## Supplementary Materials

The following are available online at https://www.mdpi.com/2073-4409/9/5/1173/s1, Figure S1: miRNAs and LncMIAT binding site. Table S1: Sequences of PCR primers used in this study, Table S2: KEGG analysis of target genes of differentially-expressed miRNAs, Table S3: KEGG analysis of target genes of miR-144. Video S1: Isolated rat primary cardiomyocytes.
