# Supplementary figures and images for: The Expression of microRNA in Adult Rat Heart with Isoproterenol-Induced Cardiac Hypertrophy
Source: Cells. 2020 May 8;9(5):1173. doi: 10.3390/cells9051173 (PMC7290591; doi:10.3390/cells9051173)

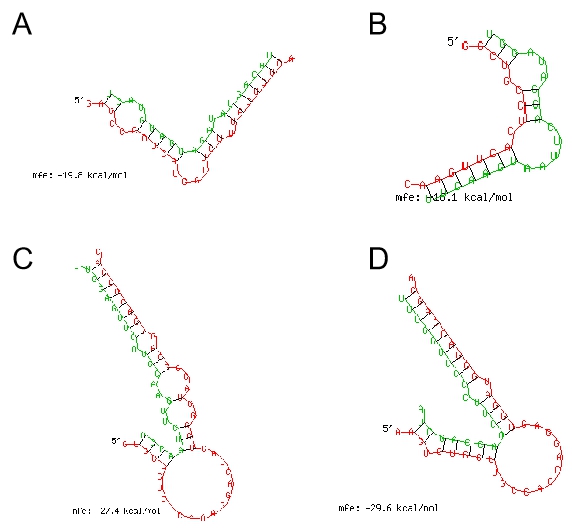


**Figure S1.** miRNAs with potential binding relationship with LncMIAT. (A) miR-144-3p. (B) miR-26b-5p. (C) miR-3068-5p. (D) miR-133a-3p.

Supplement: Supplementary file 1 [file cells-09-01173-s001.zip › Supplementary Figure S1.docx]
